# Termite antimicrobial defense through interaction with symbiotic microorganisms in nest materials

**DOI:** 10.1038/s41598-025-07667-2

**Published:** 2025-07-02

**Authors:** Masaaki Nakashima, Kenji Matsuura

**Affiliations:** Laboratory of Insect Ecology, Graduate School of Agriculture, Kyoto University, Kitashirakawa-Oiwakecho, Sakyo-ku, Kyoto, 606-8502 Japan

**Keywords:** Ecology, Behavioural ecology, Evolutionary ecology, Evolution, Social evolution

## Abstract

Social insects build robust nests to physically defend their colonies against attacks by predators and the intrusion of parasites and pathogens. While many previous studies on termite nests have focused on their physical defense functions, their nests also harbor various microorganisms that play a role in maintaining the colony’s hygienic environment. In this study, we report a dynamic defense mechanism of termite nests, where termites bury pathogen-infected corpses into the nest material, enhancing the antimicrobial defense provided by symbiotic bacteria inhabiting the nest. Termites buried pathogen-infected corpses, which could pose a high pathogenic risk, into the nest material, while they cannibalized corpses that were non-infected. In nest material where corpses were buried, the abundance of *Streptomyces*, antibiotic-producing bacteria, increased and enhanced the antifungal activity of the nest material. Furthermore, this *Streptomyces* inhibited the growth of termite pathogens and improved worker survival rates in the presence of these pathogens. These results suggest that the interaction between termites and nest-associated symbiotic bacteria, facilitated by corpse burial, contributes to the continuous maintenance of nest hygiene. This study elucidates the function of the nest as a 'living defensive wall’ and enhances our understanding of the dynamic pathogen-defense systems employed by social insects.

## Introduction

Social insects engage in a wide range of interactions with microorganisms, spanning from pathogens to mutualistic symbionts^1^. Living in dense, kin-structured colonies, social insects face unique challenges related to disease transmission, as the close proximity of individuals can facilitate the rapid spread of pathogens^2^. While this situation might appear to render these societies vulnerable, recent findings suggest that intense pathogen pressures have driven the evolution of highly effective, coordinated defenses^3,4^. Mechanisms such as social immunity enable collective behaviors within colonies to form a robust barrier against infection, thereby minimizing outbreaks and enhancing colony resilience^5^. Simultaneously, many of these insect societies have formed mutualistic partnerships with microorganisms, such as termite gut symbionts and the fungi cultivated by leaf-cutting ants, which provide essential nutritional benefits^6–8^. In addition, some species employ microbial antagonism, utilizing competitive interactions between microorganisms to suppress harmful pathogens^9–11^.

Maintaining hygiene in living environments is essential for all organisms, and the proper management of excrement and corpses is particularly critical for social insects. While most insects avoid feces, which are rich in organic material and have a high potential to act as reservoirs for pathogens^12^, termites have taken a different evolutionary path. Instead of avoiding feces, termites use their feces as building material for their nests^13^. Moreover, bacteria residing in the nest, along with antimicrobial substances derived from gut symbionts, help maintain sanitary conditions by suppressing harmful pathogens^9,14^. These microbial interactions reinforce the colony’s defense mechanisms, ensuring a hygienic environment for its members. Even more critical than managing excrement for maintaining nest hygiene is the proper handling of corpses. This is because corpses can harbor dangerous pathogens, making their removal or isolation crucial for colony health. In many social Hymenoptera, i.e., ants, bees, and wasps, behaviors like cannibalism, burial, or disposal in refuse areas are common responses to the presence of dead bodies^15,16^. Termites, however, exhibit a more sophisticated response based on the condition of the corpse. Fresh corpses may be consumed (cannibalism), but older or pathogen-infected corpses are typically buried and isolated, a behavior that serves to physically segregate the infected material from the healthy members of the colony^17,18^. This burial behavior minimizes the risk of disease transmission and contributes to the broader hygiene management strategies that are integral to the survival of social insect colonies.

**Fig. 1:**
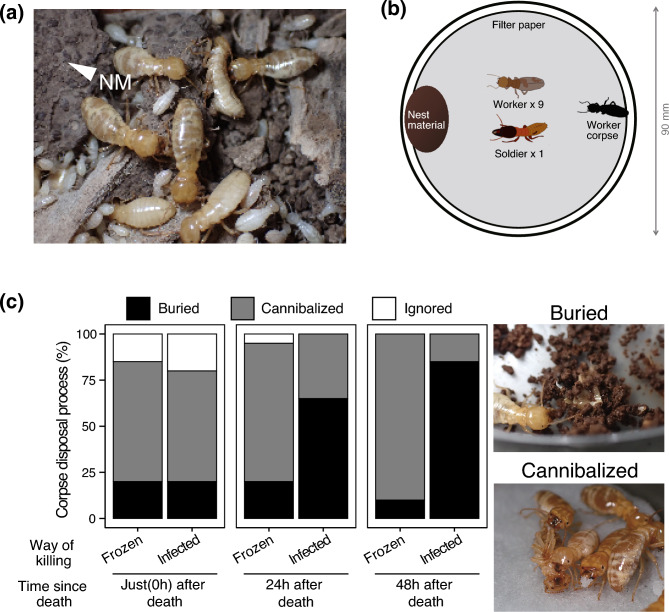
**a** The nest environment of *Hodotermopsis sjostedti*. The labeled ‘NM’ indicates nest materials constructed from termite feces. **b** Experimental setup for observing and recording corpse management behavior in *H. sjostedti* using petri dishes. **c** Comparative analysis of behavioral responses toward pathogen-infected and non-infected corpses at various postmortem times.

Dampwood termites, in particular, face significant challenges in maintaining colony hygiene due to their high-microbial-load environments. *Hodotermopsis sjostedti* (Isoptera: Archotermopsidae) is a dampwood termite distributed across East Asia, from Japan’s Satsunan Islands to northern Vietnam^19^. Colonies of *H. sjostedti*, consisting of thousands of individuals, live in the nests formed within moist, decaying wood^19,20^. They excavate tunnels that connect underground and aboveground nest sections^21^, resulting in frequent contact with soil microorganisms. In addition, they use feces as a building material (Fig. 1a), surrounding themselves with a high density of microorganisms contained within the fecal matter^22,23^. Furthermore, as a large termite species, *H. sjostedti* presents a considerable resource to pathogenic microorganisms upon death due to its large body mass, potentially creating conditions that facilitate the spread of infection within the colony. Thus, *H. sjostedti* provides an ideal opportunity to study termite hygiene maintenance systems, particularly regarding the use of feces as nest material and corpse management.

In this study, we investigated the burial behavior of corpses in *H. sjostedti* and its association with antibiotic-producing bacteria inhabiting the nest material, which is composed of feces, from the view point of nest hygiene. First, we examined the corpse management behaviors of workers in response to infected and non-infected corpses. Second, based on the observation that fungal growth is suppressed on corpses buried in the nest material, we isolated antibiotic-producing bacteria from the nest material. Third, we assessed the impact of corpse burial in the nest material on the abundance of the isolated *Streptomyces*. Finally, we evaluated the antibacterial and antifungal activity of this *Streptomyces* strain against termite pathogens and its effect on the survival of *H. sjostedti* in the presence of pathogens.

## Results

### Infected corpses were managed through burial behavior as postmortem time increased

Exposure to *Metarhizium anisopliae* spores resulted in significant mortality in *Hodotermopsis sjostedti*, highlighting the pathogenic lethality of this entomopathogenic fungus. Workers exposed to *M. anisopliae* showed significantly lower survival rates than non-infected workers, with all individuals in the infected treatment dying within 180 h, whereas only two individuals in the non-infected treatment died within the same timeframe (log-rank test, *df* = 1, χ^2^ = 218, *P* < 0.0001, Fig. S1).

Following the death of infected individuals, we investigated the burial behavior of *H. sjostedti* towards corpses with differing infection statuses and postmortem times. Corpses were categorized as “Frozen” (non-infected, prepared by freezing) or “Infected” (prepared by exposing termites to *M. anisopliae*). The burial rate of frozen corpses did not significantly change with increasing postmortem time (GLMM, likelihood ratio test, *df* = 2, χ^2^ = 0.9239, *P* = 0.6301, Fig. 1c). In contrast, the burial rate of infected corpses significantly increased as postmortem time progressed (GLMM, likelihood ratio test, *df* = 2, χ^2^ = 14.073, *P* = 0.0008, Fig. 1c). Significant differences in burial proportions were observed for infected corpses between the 0–24 h and 0–48 h postmortem times (GLMM followed by Tukey HSD, 0–24 h: *P* = 0.0135, 0–48 h: *P* < 0.001, Fig. 1c), although no significant difference was detected between the 24–48 h interval (GLMM followed by Tukey HSD, 24–48 h, *P* = 0.2741, Fig. 1c). Furthermore, at 0 h postmortem, there was no significant difference in burial proportions between infected and frozen corpses (GLMM, likelihood ratio test, *df* = 1, χ^2^ = 0, *P* = 1, Fig. 1c), but significant differences emerged at 24 and 48 h postmortem (GLMM, likelihood ratio test, *df* = 1, 24 h: χ^2^ = 7.4306, *P* = 0.0064, 48 h: χ^2^ = 9.4559, *P* = 0.0021, Fig. 1c).

### Increased symbiotic *Streptomyces* abundance and enhanced antifungal activity in buried nest materials

**Fig. 2:**
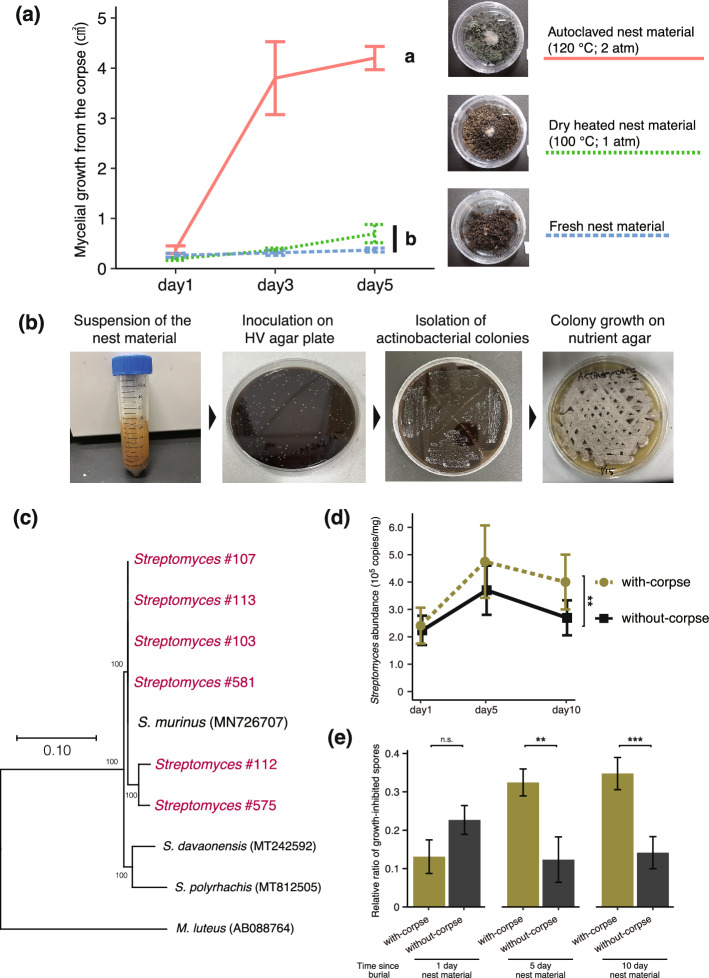
**a** Area of mycelial growth on the corpse placed on different nest materials. “Fresh” refers to untreated nest material; "Dry-heated" refers to nest material treated with dry heat (100 °C, atmospheric pressure); and “Autoclaved” refers to nest material subjected to autoclaving (120 °C, 2 atm). Significant mycelial growth was observed in the autoclaved treatment compared to both the fresh and dry-heated treatments. Different letters indicate significant differences in mycelial growth area among treatments (ANOVA, Tukey’s HSD test, α = 0.05). **b** Procedure for isolating actinobacteria from nest materials. **c** Phylogenetic tree of isolated actinomycetes, showing high sequence similarity (> 99%) to *Streptomyces murinus*. **d** Absolute abundance of *Streptomyces* actinomycetes in nest material, expressed per unit weight, and estimated by qPCR targeting the 16S rRNA gene. In the "with-corpse" treatment, pathogen-infected corpses were buried in the nest material, while in the "without-corpse" treatment, only nest material was used. Significant increases in *Streptomyces* abundance were observed on day 10 in the with-corpse treatment compared to the without-corpse treatment (GLMM, likelihood ratio test, **P < 0.01). **e** Relative ratio of growth-inhibited fungal spores based on absorbance (595 nm) in both with and without corpse treatments across three time points (day 1, 5 and 10). To eliminate the contribution of *Streptomyces* cells or growth to turbidity, nest material extracts were filtered through a 0.22 µm membrane before mixing with *Metarhizium anisopliae* spore suspensions. On days 5 and 10, the ratio of growth-inhibited spores was significantly higher in the with-corpse treatment compared to the without-corpse treatment (GLMM, likelihood ratio test, **P < 0.01, ***P < 0.001, n.s. = not significant).

We assessed the inhibitory effect of termite nest material on mycelial growth from the corpse. Mycelial growth was significantly inhibited in the fresh and dry-heat treated nest materials compared to the autoclave treatment, suggesting that these treatments preserved antifungal activities in the nest material (ANOVA, *F*_*(2,12)*_ = 153.1, *P* < 0.0001; Tukey’s HSD test, Fresh - Autoclave: *P* < 0.0001, Dry - Autoclave: *P* < 0.0001, Fresh - Dry: *P* = 0.3981, Fig. 2a). To identify the microorganisms potentially responsible for this antifungal activity, dry-heat treated samples of the nest material were suspended in sterile water and inoculated on HV agar medium (Fig. 2b). Colonies with the characteristic mycelial morphology of actinobacteria were observed, resulting in six actinobacteria isolates. Sequencing analysis revealed that all isolates from each of the six colonies showed high sequence identity (> 99%) to *Streptomyces murinus* (Fig. 2c). Among these isolates, *Streptomyces* #107 strain was selected as a representative for further experiments due to its potential role in the observed antifungal activity.

Quantification of *Streptomyces* abundance in the nest materials over time was performed by estimating the copy number of the 16S rRNA gene using qPCR on days 1, 5, and 10. The analysis compared nest materials either buried with-corpses or without-corpses. No significant difference was observed in *Streptomyces* abundance between the two treatments on days 1 and 5 (day 1: GLMM, likelihood ratio test, *df* =1, χ^2^ = 3.7412, *P* = 0.0530, day 5: GLMM, likelihood ratio test, *df* = 1, χ^2^ = 0.0107, *P* = 0.9175, Fig. 2d). On day 10, however, a significant increase in *Streptomyces* abundance was detected in the nest materials with-corpses compared to those without-corpses (GLMM, likelihood ratio test, *df* = 1, χ^2^ = 7.2431, *P* = 0.0071, Fig. 2d). For both treatments, *Streptomyces* abundance significantly increased between days 1 and 5 (GLMM followed by Tukey HSD, nest materials with corpses: *P* < 0.0001, nest materials without corpses: *P* < 0.0001, Fig. 2d). In the nest material without corpses, *Streptomyces* abundance significantly decreased between days 5 and 10 (GLMM followed by Tukey HSD, *P* = 0.0004, Fig. 2d). In contrast, no significant change was observed between days 5 and 10 in the nest material with corpses (GLMM followed by Tukey HSD, *P* = 0.9260, Fig. 2d). Additionally, in the nest material with corpses, a significant increase in *Streptomyces* abundance was observed between days 1 and 10 (GLMM followed by Tukey HSD, *P* = 0.0065, Fig. 2d), whereas no significant change was detected during the same period in the nest material without corpses (GLMM followed by Tukey HSD, *P* = 0.9327, Fig. 2d).

The antifungal activity of buried nest material was evaluated by estimating the proportion of growth-inhibited fungal spores based on turbidity measurements (absorbance at 595 nm) on days 1, 5, and 10 post-burial. To ensure that these turbidity measurements reflect the suppression of *Metarhizium anisopliae* growth rather than any contribution from Streptomyces biomass or metabolites, we used the cell-free supernatant of PBS-extracted nest materials that were further filtered through a 0.22 μm sterile membrane to remove microbial cells. On day 1, there was no significant difference in turbidity between the mixed solution of substrate extract and fungal suspension in the with-corpse and without-corpse treatments (GLMM, likelihood ratio test, *df* = 1, χ^2^ = 2.282, *P* = 0.1309, Fig. 2e). However, significant differences were observed on days 5 and 10, indicating increased antifungal activity over time in the with-corpse treatment (day 5: GLMM, likelihood ratio test, *df* = 1, χ^2^ = 10.441, *P* = 0.0012; day 10: GLMM, likelihood ratio test, *df* = 1, χ^2^ = 20.861, *P* < 0.0001, Fig. 2e). In the with-corpse treatment, significant differences in turbidity were observed between days 1 and 5 and between days 1 and 10 (GLMM followed by Tukey HSD, day1 - 5: *P* = 0.0012, day1 - 10: *P* < 0.0001, Fig. 2e), while no significant difference was detected between days 5 and 10 (GLMM followed by Tukey HSD, *P* = 0.6970, Fig. 2e). In contrast, no significant differences in turbidity were observed at any time point (days 1, 5, and 10) in the without-corpse treatment (GLMM followed by Tukey HSD, day1 - 5: *P* = 0.212, day5 - 10: *P* = 0.975, day1 - 10: *P* = 0.307, Fig. 2e). These results imply that burial of infected corpses promotes antifungal activity in the nest material over time, potentially due to the increased abundance of symbiotic *Streptomyces*.

### Antagonistic activity of *Streptomyces* against various pathogens and its protective effect on termite survival

**Fig. 3:**
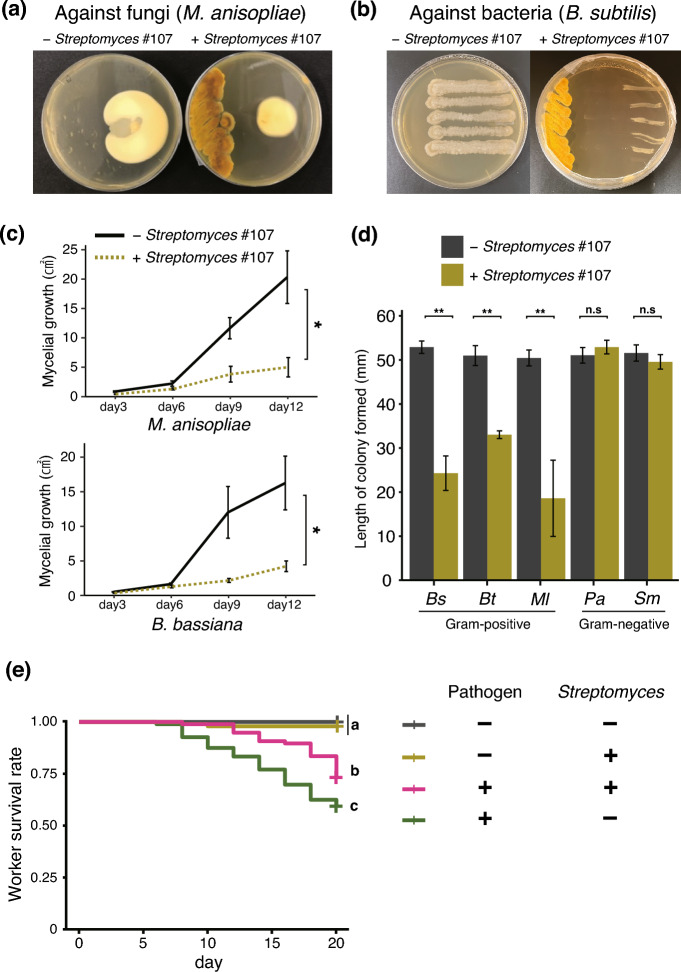
**a** Dual-culture assay results showing inhibition of fungal growth (*M. anisopliae*) by *Streptomyces* #107 strain. **b** Dual-culture assay results showing inhibition of bacterial growth (*Bacillus subtilis*) by *Streptomyces* #107 strain. **c** Inhibitory effects of *Streptomyces* on pathogenic fungi. Significant inhibition was observed against *M. anisopliae* and *Beauveria bassiana* (Wilcoxon rank sum test, *P < 0.05). **d** Inhibitory effects of isolated *Streptomyces* on Gram-positive and Gram-negative bacteria. Significant inhibition was observed against Gram-positive bacteria (*Bs* *B. subtilis*, *Bt*: *Bacillus thuringiensis*, and *Ml* *Micrococcus luteus*) compared to Gram-negative bacteria (*Pa*: *Pseudomonas aeruginosa* and *Sm* *Serratia marcescens*) (Wilcoxon rank sum test, **P < 0.01, n.s. = not significant). **e** Termite worker survival over 20 days. Pathogen (+) indicates the presence of *M. anisopliae*, while Pathogen (–) indicates its absence. *Streptomyces* (+) represents the presence of *Streptomyces* #107, and *Streptomyces* (–) represents its absence. Different letters indicate significant differences in survival rates among treatments (log-rank test, pairwise comparisons adjusted by Holm–Bonferroni method, α = 0.05).

The dual-culture antifungal assay revealed that the isolated *Streptomyces* strain #107 significantly inhibited the growth of the entomopathogenic fungi *M. anisopliae* and *B. bassiana* compared to the negative control (Wilcoxon rank sum test, against *M. anisopliae*:* P* = 0.0210, against *B. bassiana*: *P* = 0.0326, Fig. 3a, c). In the dual-culture antibacterial assay, *Streptomyces* #107 also significantly inhibited the growth of Gram-positive bacteria (*B. subtilis*, *B. thuringiensis*, and *M. luteus*) compared to the negative control (Wilcoxon rank sum test, *P* = 0.0079, Fig. 3b, d). However, no significant inhibitory effect was observed against Gram-negative bacteria (*P. aeruginosa* and *S. marcescens*) compared to the negative control (Wilcoxon rank sum test, against *P. aeruginosa*:* P* = 0.1507, against *S. marcescens*:* P* = 0.3095, Fig. 3d).

In terms of termite survival, a significant difference in survival time was observed between treatment groups (log-rank test, *df* = 3, χ^2^ = 91.6, *P* < 0.0001, Fig. 3e). Termites in the *Streptomyces* treatment showed similar survival rates to those in the control treatment (log-rank test, pairwise comparison adjusted by the Holm-Bonferroni method, *P* = 0.3, Fig. 3e). Termites exposed to *M. anisopliae* alone had significantly lower survival rates than those in the control group (log-rank test, pairwise comparison adjusted by the Holm-Bonferroni method, *P* < 0.0001, Fig. 3e). However, termites treated with both *Streptomyces* and *M. anisopliae* showed significantly higher survival rates compared to those treated with *M. anisopliae* alone (log-rank test, pairwise comparison adjusted by the Holm-Bonferroni method, *P* = 0.007, Fig. 3e). Additionally, no difference in survival time was observed among colonies (log-rank test, *df* = 3, χ^2^ = 2.6, *P* = 0.5).

## Discussion

We demonstrated that *Hodotermopsis sjostedti* suppress the growth of pathogens originating from corpses by burying them in nest materials, which are structures composed of termite feces. Interestingly, the inhibition of pathogen growth was not attributed to antimicrobial components produced by the termites themselves but rather to antimicrobial substances generated by symbiotic *Streptomyces* residing in the nest materials. These symbiotic *Streptomyces* utilize buried corpses as a nutrient source, which in turn enhances the antimicrobial activity of the nest materials. This process establishes a feedback system where higher pathogen threats and increased corpse occurrence lead to elevated concentrations of antimicrobial substances in the nest materials. These findings highlight a dynamic pathogen defense mechanism in *H. sjostedti* that relies on a mutualistic relationship with actinomycetes, rather than a static system based on termite-derived antimicrobial production.

In eusocial Hymenoptera, potential sources of infection such as feces and corpses are typically isolated in refuse piles or removed outside the nest, thereby reducing the risk of pathogen proliferation^24,25^. *H. sjostedti* uses feces as a construction material for its nests and manages infected corpses by burying them within the nest. Our study revealed that this burial behavior activates a feedback-driven defense mechanism: the progression of infections stimulates the proliferation of symbiotic *Streptomyces*, enhancing antifungal activity in nest materials. Buried corpses act as a nutrient source for *Streptomyces*, promoting the production of antimicrobial secondary metabolites. This aligns with mechanisms observed in soil bacteria, such as actinomycetes, which exhibit enhanced growth and secondary metabolite production when supplied with specific nutrients^26^. For instance, the model actinomycete *Streptomyces coelicolor A3(2)* shows increased growth and antibiotic production in chitin-rich environments^27,28^. Given that insect exoskeletons and fungal cell walls are chitin-rich, a similar nutrient-dependent mechanism likely underpins the heightened antifungal activity in the burial nest materials of *H. sjostedti*. These findings highlight how *H. sjostedti* leverages dynamic microbial processes within its nests to establish a colony-wide defense system, fortifying resistance to pathogens.

Previous studies on termite antimicrobial defenses have predominantly focused on static mechanisms, such as antimicrobial substances found in feces and saliva^29–31^. However, our findings reveal a dynamic disease-resistance framework in *H. sjostedti*, where *Streptomyces* symbionts residing in the nest materials utilize buried corpses as a nutrient source to enhance their defensive functions. Notably, *Streptomyces* has also been reported to inhabit the nest materials of various termite species^9,32,33^. For example, in several fungal-infected colonies of *Coptotermes* spp., the relative abundance of *Streptomyces* in the nest increased from 0.05% to 10%^34^. Additionally, Chouvenc et al. demonstrated in their study on *Coptotermes formosanus* that cannibalism and burial of infected corpses play a critical role in suppressing pathogen replication and preventing the spread of infection within the colony^35^. These findings not only highlight the functional role of *Streptomyces* in suppressing pathogens, but also raise broader evolutionary and ecological questions about how such associations have repeatedly emerged across phylogenetically distinct termite lineages. The recurrent detection of *Streptomyces* in the nest materials of distantly related genera, such as *Coptotermes* (Heterotermitidae) and *Hodotermopsis* (Archotermopsidae), prompts the question of whether this symbiosis represents an evolutionarily conserved trait inherited from a common ancestor or a case of convergent evolution driven by similar ecological pressures. Similar mutualisms between *Streptomyces* and social insects—such as leaf-cutting ants and beewolves—have also been reported^10,36^, suggesting that such associations may have evolved independently across multiple lineages. Comparative phylogenetic analyses of diverse insect hosts and their associated symbionts will be essential for disentangling these evolutionary scenarios. In addition to evolutionary considerations, ecological compatibility likely plays a key role in the repeated establishment and persistence of *Streptomyces* in social insect nests. Many *Streptomyces* species possess chitinase activity and can degrade chitin-rich substrates such as insect corpses and fungal cell walls^37^. The accumulation of feces and corpses in termite nests may thus create a nutrient-rich microenvironment that facilitates colonization and persistence of *Streptomyces*. Supporting this idea, previous studies have shown that termite nest substrates can sustain stable populations of *Streptomyces*^9^. Such ecological conditions may promote the stable acquisition and long-term retention of these bacteria by *Hodotermopsis* termites. In the present study, *Streptomyces murinus* was consistently isolated from the nest materials of multiple *H. sjostedti* colonies. This recurring pattern suggests either selective acquisition from a diverse environmental pool or vertical maintenance of a specific strain. In *C. formosanus*, *Streptomyces* was rarely detected in alates or founding nests and was shown to be acquired primarily from surrounding soil rather than through vertical transmission^38^. *H. sjostedti* is a multiple-site nester that constructs colonies across several pieces of damp wood connected by subterranean tunnels^21^, thereby maintaining contact with the surrounding soil. This ecological feature suggests that *S. murinus* may be horizontally acquired from soil after colony establishment, rather than transmitted vertically. Future studies should adopt experimental approaches similar to those used by Chouvenc et al.^38^, including comparative analyses of symbiotic actinomycetes between parental and incipient colonies, to determine the transmission mode of *Streptomyces* in this species.

Symbiotic relationships between social insects and microorganisms are well-documented, particularly regarding the role of symbiotic microbes in defending against pathogens through antagonistic interactions among microorganisms within their nest^1,39^. For example, leaf-cutting ants cultivate *Leucoagaricus* fungi in their fungal gardens as part of a nutritional mutualism^40,41^. These gardens, however, are vulnerable to *Escovopsis*, a specialized pathogen that compromises fungal health and colony survival^42,43^. In response, leaf-cutting ants employ diverse strategies to suppress *Escovopsis*, including meticulous cleaning of fungal gardens, secretion of antimicrobial substances from their metapleural glands, and the antagonistic actions of *Pseudonocardia* bacteria residing on their cuticle^44,45^. Additionally, *Streptomyces* species within ant nests produce antibiotics such as candicidin, which specifically inhibit *Escovopsis* without harming the mutualistic *Leucoagaricus* fungi^10^. These microbial defenses have been recognized as an extended disease resistance mechanism, operating largely independently of their social insect hosts. Our study highlights that antagonistic defenses mediated by symbiotic microbes can be dynamically shaped by the behaviors of social insects, resulting in an interactive and adaptive defense mechanism. This perspective frames defensive symbioses as dynamic antimicrobial systems, emphasizing the reciprocal and behavior-driven interactions between social insects and their microbial partners. Such a framework broadens our understanding of symbiotic relationships and may have far-reaching implications for a wide range of social insects and their associated microbiota, showcasing the intricate interplay that enhances collective disease resistance.

The feedback-driven antimicrobial mechanism observed in *H. sjostedti* demonstrates how burial behavior not only mitigates pathogen threats but actively enhances the disease resistance of the nest environment. This study reveals the remarkable capacity of symbiotic microbes to adaptively respond to environmental stimuli triggered by termite behaviors, providing new insights into the co-evolution of social insects and their microbial partners. More broadly, our results add to the growing body of evidence that microbial symbiosis is integral to the ecological success of social insects. Unlike static antimicrobial systems reliant on host-derived substances, the dynamic and behavior-driven microbial defense observed in *H. sjostedti* serves as a model for understanding how social insects leverage symbiotic relationships to address complex pathogen challenges. This framework likely extends beyond termites to other social insect taxa. Future research should investigate the prevalence and universality of such dynamic microbial defenses across diverse termite species and social insects. Long-term studies focusing on the interplay between host behavior, microbial communities, and environmental conditions will be vital to uncovering the evolutionary and ecological significance of these relationships.

## Materials and methods

### Termite

Colonies of *Hodotermopsis sjostedti* were collected from Amami-Oshima Island, Kagoshima Prefecture, Japan (colonies KM103, KM107, KM112, KM113, KM240, KM244, KM269, MT675 and MT681). Termites were extracted by dissecting decayed wood, placed in plastic containers (35 cm × 25.5 cm × 6 cm) lined with a brown-rotted pinewood mixed cellulose (BPC) medium^46^ and pine blocks, and the colonies were kept in darkness at 25 °C.

### Termite pathogen

We applied *Metarhizium anisopliae*, which is commonly used as a model entomopathogen^47^, in our bioassays. The pathogenicity of *M. anisopliae* (NBRC 31961), provided by the Biological Resource Center (National Institute of Technology and Evaluation), was tested on individual termites. We initially investigated the pathogenic lethality of this *M. anisopliae* strain against *H. sjostedti*. Spores of *M. anisopliae* were cultured on potato dextrose agar (PDA) and incubated at 28 °C in the dark. After 14 days, spores were harvested from the plates using a 0.05% Tween 80 solution, and a stock suspension of 1.0 × 10^6^ spores/mL was prepared through serial dilutions with the help of a hemocytometer. Termites were individually placed in 24-well plates lined with filter paper, and 20 μL of the spore suspension was added to each well for the treatment group, while a 0.05% Tween 80 solution without spore was used for the control group. Termites were monitored every 12 h, and their survival time was recorded. After death, termites were stored at – 20 °C for use in subsequent experiments. The experiment involved two treatments (with and without *M. anisopliae*), four experimental blocks (four colonies each), and 24 individual replicates per treatment, totaling 192 experimental units.

### Behavioral response toward infected and non-infected corpses with different post-mortem times

The response of termites to fungal-infected and non-infected corpses was assessed using a petri dish assay. Termites from the same colony, matched for similar size, were randomly selected for the experiments. Infected corpses were obtained from previous mortality experiments, whereas non-infected corpses were prepared by freezing. Both types of corpses were placed in petri dishes (90 × 15 mm) at 25 °C and observed at post-mortem intervals of 0, 24, and 48 h. Each dish, lined with moistened filter paper, contained 2.0 g of colony-derived nest material placed along the edge, with the corpse positioned on the opposite side (Fig. 1b). Nine workers and one soldier termite were introduced to each dish. After 24 h, corpse management behaviors were classified into three visually distinguishable, non-overlapping categories:

Cannibalized: Focal corpse is being bitten by nestmates and its body is no longer intact.

Buried: Focal corpse has been covered with pieces of feces or nest materials.

Ignored: Focal corpse is intact, and unburied, with no interaction from nestmates.

The experiment included six treatments (infected or non-infected corpses at different post-mortem intervals), with two experimental blocks (one per colony) and 10 replicates per treatment, resulting in a total of 120 experimental units.

### Comparison of mycelial growth from buried corpses in differently treated nest materials

Three treatment groups were prepared to assess the antifungal activity of the nest material: a fresh group, where the nest material was left untreated (Fresh); a dry heat sterilization group (100 °C, atmospheric pressure, 30 min), to remove microorganisms other than heat-resistant actinobacteria (Dry); and an autoclave group (120 °C, 2 atm, 20 min), to eliminate most microorganisms (Autoclave). The treated nest material was divided into 2.0 g portions and placed in sterile petri dishes (30 × 15 mm). In the center of each nest materials, a worker, killed by decapitation, was placed. Mycelial growth from the corpse was recorded. The petri dishes were wrapped in two layers of Parafilm and incubated at 25 °C for 5 days. Five replicates were prepared for each treatment. The size of the fungal colony was measured every 2 days by photographing the dish with a digital camera (TG-6; Olympus, Tokyo, Japan), and the fungal colony area was calculated using ImageJ software^48^.

### *Streptomyces* actinobacteria isolation from the nest materials

Nest materials of six *H. sjostedti* colonies were collected from each breeding cases in the laboratory. To isolate actinobacteria from termite nest material, we followed the previous protocols^49^. Fresh nest material samples (six termite colonies in total) were subjected to dry heat treatment at 100 °C for 30 min to pre-treat the material. This step was performed to account for the high resistance of most actinobacteria spores to both dry and wet heat^50^. Suspension of dry-heated nest materials were prepared in sterile distilled water and inoculated onto plates of humic acid vitamin (HV) agar medium^51^, and incubated in the dark at 28 °C for 10 days. Colonies with the morphology of actinobacteria were selected for subculturing. Pure cultures were inoculated on ISP2 agar media, and this agar plug were stored in a 10% glycerol solution at – 80 °C. To do the molecular identification for the isolated actinobacteria from the nest material, we extracted DNA using NucleoSpin® Microbial DNA kit (Takara, Shiga, Japan) according to the manufacturer’s protocol. The 16S rRNA sequence was obtained by primer pair (10f.: 5ʹ- GTTTGATCCTGGCTCA-3ʹ, 800r: 5ʹ-TACCAGGGTATCTAATCC-3ʹ). The total PCR volume was 20 μL, including 10 μL of KOD One^®^ PCR Master Mix (TOYOBO, Osaka, Japan), 0.6 μL of each primer (10 μM), 1 μL of DNA template and 7.8 μL of nuclease free water. A ProFlex PCR System (Applied Biosystems, MA, USA) was used for PCR amplification, and the amplification procedure was as follows: 30 cycles of 98 °C (10 s), 55 °C (5 s) and 68 °C (5 s). The PCR products were confirmed by electrophoresis on a 1.5% agarose gel, and the target PCR product was sequenced by using the BigDye Terminator version 3.1 Cycle Sequencing Kit and an ABI 3500 Genetic Analyzer (both from Applied Biosystems, CA, USA). The 16S rRNA sequence was sent for BLAST in NCBI. Based on the hits from the BLAST search, a phylogenetic tree of the identified bacteria was generated with MEGA 11 software^52^. The maximum likelihood method was used to construct a phylogenetic tree based on the 16S rRNA sequences, and the phylogenetic tree was evaluated with bootstrap analysis. Sequences were deposited in GenBank database under the accession numbers LC858664–LC858669.

### Quantification of *Streptomyces* abundance in buried nest materials

We quantified *Streptomyces* in the nest materials with and without infected corpses by qPCR. 100 μL of *M. anisopliae* suspension was added to 12-well plates lined with filter paper, and worker individuals were placed in the wells to expose them to the pathogen. Workers were kept at 25 °C until death by infection, checked for death every 12 h, and dead individuals were collected and frozen at – 20 °C until used in the next experiment as the infected corpses. The same nest material as that of the worker was collected from each colony (2.0 g), the "with-corpse” treatment with the nest material buried the infected corpse, and the "without-corpse” treatment with only the nest material as a control treatment, were prepared. We prepared three replications from each of the three colonies. After 1, 5 and 10 days of incubation at 25 °C, respectively, DNA was extracted from each nest material and used for quantification. In order to estimate differences in absolute abundance of *Streptomyces*, qPCR analysis was performed. Quantification from crudo samples with DNA extraction from nest material was conducted by modifying previous methods^53^. To extract DNA from the weighed nest materials, the Fast DNA SPIN Kit (Funakoshi, Tokyo, Japan) was used according to the manufacturer’s protocol. Nest material samples were retrieved from three colonies each. For quantitative evaluation by qPCR, *Streptomyces* specific primer sets (StrepB: 5'-ACAAGCCCTGGAAACGGGGT-3'; StrepF: 5'-ACGTGTGCAGCCCAAGACA-3') targeting the 16S rRNA gene^54^ were used. Since the target sequence of qPCR is expected to be around 1k bp in this experiment, KOD SYBR^™^ qPCR Mix (TOYOBO, Osaka, Japan), which is suitable for long targets, was used. Extracted DNA was used in duplicate for each sample of the nest material, and diluted to within 80 ng per 20 µL reaction in accordance with qPCR reagent specifications. All reactions were set up in a volume of 20 μL, including 10 μL of KOD SYBR^™^ qPCR Mix, 2 μL each of 2 μM forward and reverse primers, 4.6 μL of nuclease free water, 0.4 μL ROX reference dye and 1 μL of DNA template. The reactions were amplified using the StepOnePlus real-time PCR system (Applied Biosystems, MA, USA) with the following parameters: 98 °C (2 min) followed by 45 cycles of 98 °C (10 s), 55 °C (10 s), and 68 °C (1min 30 s). The threshold cycle of each sample was determined during the exponential phase of amplification. After the PCR, a melting curve was constructed in the 60–99 °C range. The absolute amount of *Streptomyces* was determined by reference to a standard curve as the amount of *Streptomyces* per mg based on the nest material sample weight used for extraction. To determine a standard curve, DNA was extracted from monocultured *Streptomyces* #107 as a representative, and primer sets of StrepB and StrepF were used to confirm the specificity of amplification by PCR. The amplified fragments were then ligated into pGEM T-easy vector (Promega, Madison, WI) and introduced into *E. coli* JM109 Competent Cells (Takara Bio, Shiga, Japan) by heat shock. The transfected colonies were selected by blue/white screening and colony PCR, and incubated in 10 mL of LB liquid medium supplemented with 10 μL of ampicillin (100 mg/mL) for 16 h at 37 °C and 120 rpm with shaking. Plasmids were purified from the cultures by Plasmid Easy Pure (Qiagen, Hilden, Germany), and four different plasmid concentrations were obtained by tenfold dilution of the purified products.

### Quantification of antifungal activity in buried nest materials

The antifungal activity of the nest materials with and without infected corpses were determined by modifying the previous method^55,56^ and measuring the reduction of *M. anisopliae* blastospores based on absorbance. With-corpse and without-corpse nest materials were the same as those used for *Streptomyces* quantification at day 1, day 5, and day 10. For each replicate of each treatment type consisting of 0.25 g of nest material for antifungal activity measurements, the nest material was crushed in a centrifuge tube on ice and dissolved in phosphate buffered saline (PBS) at a ratio of 2 μL of PBS for 1 mg of nest material weight. As the spores-growth control, the autoclaved nest material was crushed in a centrifuge tube on ice and dissolved in PBS at a ratio of 2 μL of PBS for 1 mg of nest material weight. Then, the homogenates were centrifuged at 6000 × g for five minutes at 4 °C and then 100 μL of the extract supernatants were centrifuged at 6000×g for five minutes at 4 °C again. Extract supernatants were centrifuged at 10,000×g for five minutes at 4 °C using a 0.22 µm centrifugal filter (Ultrafree-MC GV 0.22 μm; Merck) to sterilize. Then, 20 μL of the supernatants were extracted and stored at – 80 °C until antifungal activity assay. When measuring antifungal activity, we used 96-well microplates with 50 μL sabouraud dextrose broth (SDB), 2 μL blastospores (1.0 × 10^6^ spores/mL), 2 μL supernatant per well. Additionally, we used 50 μL SDB, 2 μL the blastospores, 2 μL autoclaved supernatant per well for spores-growth control, and 50 μL SDB, 2 μL PBS, 2 μL autoclaved supernatant per well for standards. After 72 h of cultivation in constant temperature shaker (300 rpm; 25 °C), the absorbance of each well was measured by the microplate spectrophotometer (Multiskan FC; Thermo Scientific, USA) at a wavelength of 595 nm. For each treatment and time point, three replicates were taken from three colonies, with each well measured in triplicate, resulting in three measurements for each plate.

### Antagonistic effect of *Streptomyces* against various microorganisms

The *Streptomyces* isolate obtained from the *H. sjostedti* nest material was tested for its antifungal activity against two fungal entomopathogens, *M. anisopliae* (NBRC 31961) and *Beauveria bassiana* (NBRC 103721), provided by the Biological Resource Center (National Institute of Technology and Evaluation). Additionally, the *Streptomyces* isolate was tested against Gram-negative and Gram-positive bacteria to determine a basic profile of their overall antibacterial activity. Test species included *Bacillus subtilis* (NBRC 3009), *Bacillus thuringiensis* (NBRC 13865), *Micrococcus luteus* (NBRC 16250), *Pseudomonas aeruginosa* (NBRC 3080), and *Serratia marcescens* (Rs 200308G8). The strain of *S. marcescens* used in this bioassay was isolated from *Reticulitermes speratus* bodies following a previously described procedure^14^. Bacterial cells were spread on LB medium and incubated at 28 °C in the dark. After 24 h of incubation, bacterial colonies were collected from these plates using a 0.05% Tween 80 aqueous solution for bacterial suspension, and stock suspensions of 1.0 × 10^8^ CFU/mL were prepared by the plate dilution method. These suspensions were stored at 4 °C and used for dilutions with sterile deionized water within 15 days of the experiments described.

For antimicrobial screening, a dual-culture assay was performed according to the protocol as described previously^57^. The *Streptomyces* isolate was individually inoculated on ISP2 media along the edge of the agar to allow diffusion of secondary metabolites through the plate over 5 days. Subsequently, a 7 mm diameter plug of fungal culture was placed on the opposite side of the *Streptomyces* inoculation. For bacteria, a bacterial suspension was streaked in a line approximately 5 cm long. Each test was performed with 5 replicates. Incubation for each test bacterium was carried out at 28 °C. Growth inhibition measurements were taken 24 h after overlay for indicator bacteria and every 3 days after overlay for indicator fungi.

### Effect of the *Streptomyces* on the survival of termite in the nest-like nutritional environment

To determine the direct effect of exposure of *Streptomyces* #107 strain to entomopathogens on termite survival, individual termites were reared for 20 days on a medium with nutrient requirements equivalent to those of termite nest material and the number of deaths was recorded every 2 days. Tests were performed in 24-well plates using a modification of a previously described method^9^. To simulate the nest-like nutritional environment, autoclaved nest material was added 15% with brown-rotted pinewood mixed cellulose medium^46^, a termite’s food source, and each well was filled as the nest medium. To prepare microbial suspensions, spores of *M. anisopliae* or *Streptomyces* #107 strain were spread on ISP2 medium and incubated in the dark at 28 °C. After the inoculated plates were incubated for 14 days, fresh spores were collected from these plates in 0.05% Tween 80 solution (for spore suspension) and stock suspensions of 1.0 × 10^8^ spore/mL (for *M. anisopliae*) or 1.0 × 10^8^ CFU/mL (for *Streptomyces*) were prepared by serial dilution. In the *Streptomyces* only treatment and in the *Streptomyces* and *M. anisopliae* mixed treatment, 0.2 ml of 0.05% Tween 80 solution containing *Streptomyces* #107 was added to the nest medium. This treatment simulated a termite nest material environment in which *Streptomyces* community had already formed. An equal volume (0.2 ml) of 0.05% Tween 80 solution was added to the control treatment wells (without *Streptomyces* #107 and *M. anisopliae*) and the *M. anisopliae* treatment wells. The 24-wells with the mixture were stored in the dark at 25 °C for 10 days. Finally, after 10 days of incubation, each well was given 0.2 ml of 0.05% Tween 80 solution or 0.2 ml of *M. anisopliae* suspension in 0.05% Tween 80 solution, depending on treatment. One *H. sjostedti* worker was introduced to each well. The 24-wells were sealed with parafilm, and kept at 25 ℃ in the dark. Worker mortality was recorded every 2 days. The experiment consisted of four treatments (*Streptomyces* treatment, *M. anisopliae* treatment, *Streptomyces* and *M. anisopliae* mixed treatment, and control treatment), with four experimental blocks (one block per colony) and 24 replicates per treatment, totaling 384 experimental units.

### Statistical analysis

All statistical analyses were performed using R software (version 4.2.1)^58^, with the survival package for Kaplan–Meier analysis and the lme4 package for generalized linear mixed models (GLMM). Kaplan–Meier survival analysis was conducted to examine the pathogenicity of the entomopathogenic fungus *M. anisopliae* against *H. sjostedti* workers. The survival analysis included 192 experimental units derived from four colonies, with 24 replicates per treatment (with and without *M. anisopliae*). Survival distributions were compared using log-rank tests. A GLMM with a binomial error distribution and logit link function was applied to compare the frequency of burial behavior exhibited by colony members under different corpse conditions. The analysis included 120 experimental units derived from two colonies, with 10 replicates per treatment at each of three postmortem time intervals (0, 24, and 48 h). The response variable was the number of buried corpses per analyzed corpse management behavior, with corpse condition and postmortem time as fixed effects, and colony as a random effect.

For the mycelial growth assay, ANOVA was used to test whether the mycelial growth area from buried corpses differed among nest material treatments. The analysis included 15 experimental units derived from three treatments (fresh, dry-heat, and autoclave), with five replicates per treatment. Tukey’s HSD test was employed for post hoc comparisons of mean mycelial growth area across treatments. To investigate whether corpse presence affects Streptomyces abundance, a GLMM assuming a gamma distribution was used, with time and 16S rRNA gene copy numbers as response and explanatory variables, respectively. This analysis included 54 experimental units, derived from three colonies and three time points (days 1, 5, and 10), with three replicates per treatment (with and without corpses) at each time point. Colony was included as a random factor. Likelihood ratio tests were conducted, and Tukey-adjusted pairwise comparisons were applied for multiple comparisons. Similarly, the effect of corpse presence on the antifungal activity of nest material was evaluated using a GLMM with a gamma distribution, absorbance (wavelength = 595 nm) as the response variable, and corpse presence and burial time as fixed effects. The analysis included 54 experimental units, derived from three colonies and three time points (days 1, 5, and 10), with three replicates per treatment (with and without corpses) at each time point. Colony was treated as a random factor, and likelihood ratio tests followed by Tukey-adjusted pairwise comparisons were conducted.

For the antifungal and antibacterial activity assays of isolated *Streptomyces*, a Wilcoxon rank-sum test was conducted to evaluate differences in fungal growth area and bacterial colony length between treatments with and without *Streptomyces*. These assays consisted of 10 experimental units, comprising two treatments (with and without *Streptomyces*) with five replicates per treatment. In the bioassay assessing the effect of *Streptomyces* on the survival of *H. sjostedti* workers, Kaplan–Meier survival analysis was employed. The analysis included 384 experimental units, derived from four colonies, with 24 replicates per treatment across four treatments (*Streptomyces* only, *M. anisopliae* only, *Streptomyces* + *M. anisopliae*, and control). Survival distributions were compared using log-rank tests, with Holm-Bonferroni correction for multiple comparisons.

## Supplementary Information

Supplementary Information 1.

Supplementary Information 2.

## Data Availability

The dataset supporting the conclusions of this article is included within the article and its additional file.
