# Supplementary material for: Termite antimicrobial defense through interaction with symbiotic microorganisms in nest materials
Source: Sci Rep. 2025 Jul 2;15:23391. doi: 10.1038/s41598-025-07667-2 (PMC12222720; doi:10.1038/s41598-025-07667-2)
Supplement: Supplementary file 2 — Supplementary Information 2. [file 41598_2025_7667_MOESM2_ESM.pdf]

**Supplementary information**

# Termite antimicrobial defense through interaction with symbiotic microorganisms in nest materials

Masaaki Nakashima[1], Kenji Matsuura[1*]

[1]Laboratory of Insect Ecology, Graduate School of Agriculture, Kyoto University, Kitashirakawa-Oiwakecho, Sakyo-ku, Kyoto 606-8502, Japan

ORCID No.
Masaaki Nakashima (0009-0003-8690-4421), Kenji Matsuura (0000-0002-9099-6694)

*Corresponding Author:
Kenji Matsuura (Email: matsuura.kenji.6s@kyoto-u.ac.jp)

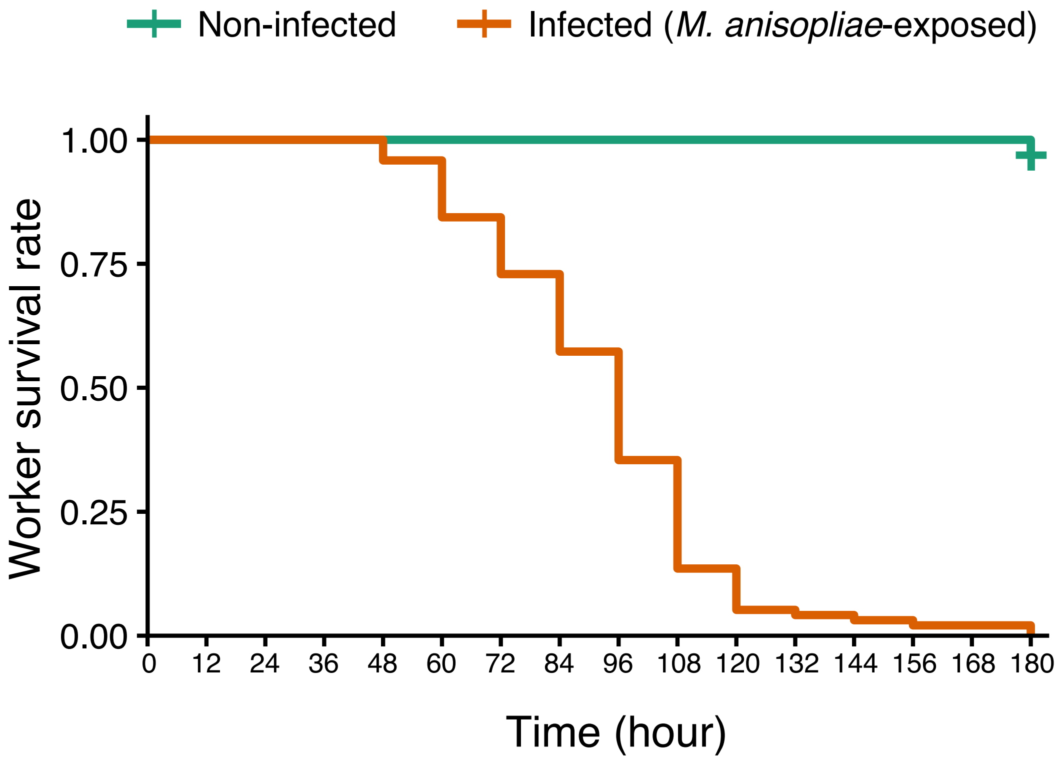


**Supplementary figure. S1**

Kaplan-Meier survival analysis of termite workers exposed to the entomopathogenic fungus *Metarhizium anisopliae* compared to non-infected workers. Workers exposed to *M. anisopliae* showed significantly reduced survival times (log-rank test, $df = 1$, $\chi^2 = 218$, $P < 0.0001$).
